# Social contagions on interdependent lattice networks

**DOI:** 10.1038/srep44669

**Published:** 2017-03-16

**Authors:** Panpan Shu, Lei Gao, Pengcheng Zhao, Wei Wang, H. Eugene Stanley

**Affiliations:** 1 School of Sciences, Xi’an University of Technology, Xi’an, 710054, China; 2 Web Sciences Center, University of Electronic Science and Technology of China, Chengdu, 610054, China; 3 School of Physics and Optoelectronic Engineering, Xidian University, Xi’an, 710071, China; 4 Big data research center, University of Electronic Science and Technology of China, Chengdu 610054, China; 5 Center for Polymer Studies and Department of Physics, Boston University, Boston, Massachusetts, 02215, USA

## Abstract

Although an increasing amount of research is being done on the dynamical processes on interdependent spatial networks, knowledge of how interdependent spatial networks influence the dynamics of social contagion in them is sparse. Here we present a novel non-Markovian social contagion model on interdependent spatial networks composed of two identical two-dimensional lattices. We compare the dynamics of social contagion on networks with different fractions of dependency links and find that the density of final recovered nodes increases as the number of dependency links is increased. We use a finite-size analysis method to identify the type of phase transition in the giant connected components (GCC) of the final adopted nodes and find that as we increase the fraction of dependency links, the phase transition switches from second-order to first-order. In strong interdependent spatial networks with abundant dependency links, increasing the fraction of initial adopted nodes can induce the switch from a first-order to second-order phase transition associated with social contagion dynamics. In networks with a small number of dependency links, the phase transition remains second-order. In addition, both the second-order and first-order phase transition points can be decreased by increasing the fraction of dependency links or the number of initially-adopted nodes.

Real-world networks are often interdependent and embedded in physical space^1^,^2^,^3^,^4^. For example, the world-wide seaport network is strongly coupled to the world-wide airport network, and both are spatially embedded^5^. The nodes in a communications network are strongly coupled to the nodes in the power grid network and both are spatially embedded^2^. The Internet is a network of routers connected by wires in which the routers are grouped as autonomous systems (AS), and at this level the Internet itself can be seen as a set of interconnected AS embedded in physical space^1^.

We know that these interdependent spatial networks can significantly influence the dynamical processes in them^3^,^4^,^6^,^7^,^8^,^9^,^10^. The percolation transition can change from discontinuous to continuous when the distance in space between the interdependent nodes is reduced^11^, and the system can collapse in an abrupt transition when the fraction of dependency links increases to a certain value^12^. The universal propagation features of cascading overloads, which are characterized by a finite linear propagation velocity, exist on spatially embedded networks^13^. In particular, a localized attack can cause substantially more damage to spatially embedded systems with dependencies than an equivalent random attack^14^. Spatial networks are typically described as lattices^15^,^16^. Studies of the dynamics in interdependent lattices have found that asymmetric coupling between interdependent lattices greatly promotes collective cooperation^17^, and the transmission of disease in interconnected lattices differs as infection rates differ^18^. Recent works demonstrated a change in the type of phase transition on related social dynamics in interdependent multilayer networks^19^,^20^,^21^,^22^. Systematic computations revealed that in networks with interdependent links so that the failure of one node causes the immediate failures of all nodes connected to it by such links, both first- and second-order phase transitions and the crossover between the two can arise when the coupling strength is changed^23^. The results of ref. 24 demonstrated that these phenomena can occur in the more general setting where no interdependent links are present.

Social contagions^25^,^26^,^27^,^28^,^29^,^30^, which include the adoption of social innovations^31^,^32^,^33^, healthy behaviors^34^, and the diffusion of microfinance^35^, are another typical dynamical process. Research results show that multiple confirmations of the credibility and legitimacy of a piece of news or a new trend are ubiquitous in social contagions, and the probability that an individual will adopt a new social behavior depends upon previous contacts, i.e., the social reinforcement effect^34^,^36^,^37^,^38^,^39^. A classical model for describing the reinforcement effect in social contagions is the threshold model^40^ in which an individual adopts the social behavior only if the number or fraction of its neighbors who have already adopted the behavior exceeds an adoption threshold. Using this threshold model, network characteristics affecting social contagion such as the clustering coefficient^41^, community structure^42^,^43^, and multiplexity^44^,^45^,^46^ have been explored, but the existing studies paid little attention to the dynamics of social contagion on interdependent spatial networks.

Here we numerically study social contagion on interdependent spatial networks using a novel non-Markovian social contagion model. A node adopts a new behavior if the cumulative pieces of information received from adopted neighbors in the same lattice exceeds an adoption threshold, or if its dependency node becomes adopted. We compare the dynamics of social contagion in networks when we vary the fraction of dependency links and find that the density of final recovered nodes increases greatly in networks when the number of dependency links is high. We also find that the fraction of dependency links can change the type of the phase transition. We use a finite-size analysis method^47^ to identify the type of phase transition and find that the phase transition is second-order when the fraction of dependency links is small and first-order when the fraction is large. In interdependent spatial networks the fraction of initially-adopted nodes *ρ*_0_ may also affect the phase transition. Concretely, when we increase *ρ*_0_ the type of phase transition does not change in networks with a small fraction of dependency links, but changes from first-order to second-order in networks with a large fraction of dependency links. The phase transition points decrease when the fraction of dependency links or initially-adopted nodes increases.

## Results

### Non-Markovian social contagion model on interdependent spatial networks

**Figure 1:**
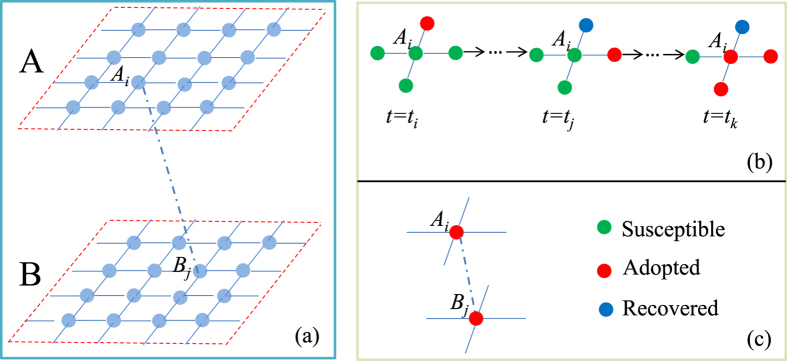
Illustration of the social contagion on the interdependent spatial network. (**a**) Interdependent spatial network composed of two 2-dimensional periodic square lattices *A* and *B*, where a node *A*_*i*_ in lattice *A* is randomly interconnected with a node *B*_*j*_ in lattice *B*. (**b**) Connected propagation with *T* = 3: In lattice *A*, the node *A*_*i*_ becomes adopted after exposing three times to the social behavior from its adopted neighbors. Here *t*_*i*_, *t*_*j*_ and *t*_*k*_ are any three different time steps of the dynamics confined with *t*_*i*_ < *t*_*j*_ < *t*_*k*_. (**c**) Dependency propagation: At some step the node *B*_*j*_ becomes adopted, and then the corresponding dependency node *A*_*i*_ adopts the social behavior.

Our spatial network model consists of two identical two-dimensional lattices *A* and *B* of linear size *L* and *N* = *L* × *L* nodes with periodic boundaries, as shown in Fig. 1(a). In each lattice, *p* fraction of nodes are randomly chosen as dependency nodes with two types of link, connectivity links (i.e., links between two nodes in the same lattice) and dependency links (i.e., links between nodes in lattice *A* and nodes in lattice *B*). The remaining 1 − *p* fraction of nodes only have connectivity links. More details of the interdependent spatial networks can be found in the Method section.

We divide the interdependent network population into three compartments, susceptible (S), adopted (A), and recovered (R) nodes. We generalize the cascading threshold model^40^ to the interdependent spatial network, describe the dynamics of social contagion using the susceptible-adopted-recovered (SAR) model, and add social reinforcement through considering individual memory. Within the same lattice, nodes can retain their memory of previous information received from neighbors and adopt the new behavior if the cumulative pieces of information received from their neighbors exceeds an adoption threshold *T* [see Fig. 1(b)]. We designate this type of behavior adoption *connected infection*. A node can also adopt the new behavior when its corresponding dependency node becomes adopted. We designate this type of behavior adoption *dependency infection* [see Fig. 1(c)].

The simulations of the social contagion dynamics are implemented by using synchronous updating methods^48^. Initially, *ρ*_0_ fraction of nodes are randomly selected to be adopted (i.e., to serve as seeds) in lattice *A*, and we leave all other nodes in the susceptible state. Each node has a record *m*_*i*_ of the pieces of received information from its neighbors. Initially, *m*_*i*_ = 0 for every node. At each time step, each adopted node transmits the behavior information to its susceptible neighbors in the same lattice with probability *λ* through the connectivity links. Once a susceptible node *i* is exposed to the information from an adopted neighbor, its *m*_*i*_ increases by one. If *m*_*i*_ is greater than or equal to the adoption threshold *T*, the susceptible node *i* will become an adopted node (Here connected infection happens). Once node *i* becomes an adopted one, its susceptible dependency nodes also become adopted at the same time (Here dependency infection happens). Infected nodes may also lose interest in the social behavior and become recovered with a probability *u*. When an adopted node becomes a recovered node it no longer takes part in the propagation of the social behavior. The time step is discrete and increases by Δ*t* = 1. The dynamics of social contagion evolve until there are no more adopted nodes in the interdependent spatial network. In this paper, *T* is set to 3, unless otherwise specified. Note that our model is similar to the susceptible-infected-recovered (SIR) epidemic model^49^,^50^ but differs in that we add the memory of received information^34^,^35^,^36^,^47^,^51^,^52^. Our proposed model of social contagion may describe the adoption of real-world social behavior. For example, a couple can discuss household products they use with their circle of friends. A wife or husband may adopt a new product if many of their friends have adopted it, or if either wife or husband adopts it then the other immediately adopts it as well.

### Effects of the fraction of dependency links

**Figure 2:**
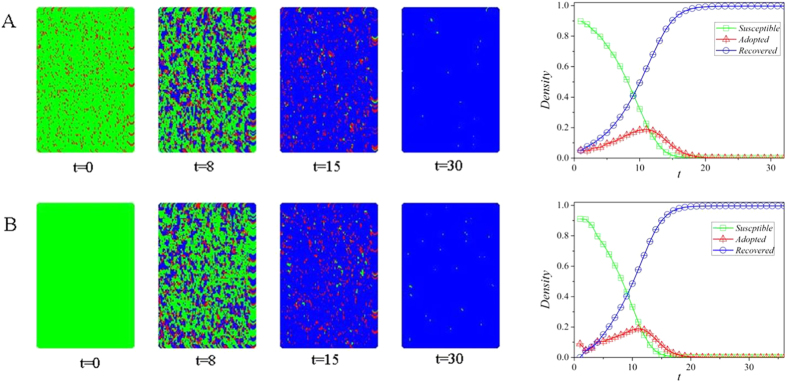
Spatio-temporal pattern of the dynamical process and time evolution of the population densities on interdependent spatial networks. The paraments are chosen as *N* = 10^4^, *p* = 0.9, *ρ*_0_ = 0.1, *λ* = 0.8, *μ* = 0.5, and *T* = 3. The colors green, red and blue represent susceptible, adopted and recovered states, respectively.

Figure 2 shows a plot of the spatio-temporal pattern of the dynamical process at different stages. At *t* = 0 each node is either susceptible or adopted. After several steps (e.g., *t* = 8) susceptible, adopted, and recovered nodes can co-exist. As *t* increases (e.g., *t* = 15 and *t* = 30) recovered nodes gradually dominate. Figure 2 also shows the time evolution of the population densities in which the density of susceptible (recovered) nodes decreases (increases) with time and ultimately reaches some value. The density of the adopted individuals decreases initially due to the fact that the number of individuals who newly adopt the behavior is less than that of individuals who become recovered. Then it is advanced with the growth of newly adopted individual and reaches the maximum value at *t* ≈ 12.

**Figure 3:**
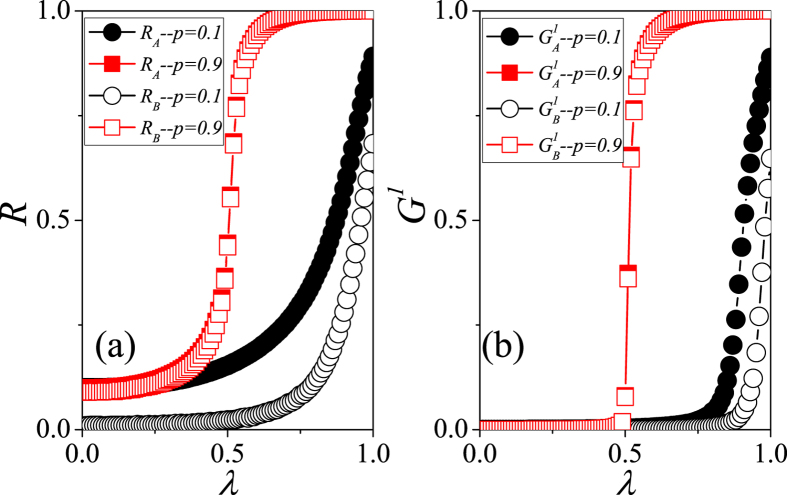
Comparison of the average outbreak size *R* and the giant connected components of recovered nodes *G*_1_ among different interdependent spatial networks. (**a**) *R*_*A*_ and *R*_*B*_ vs. *λ* for *p* = 0.1 (solid and empty circles) and *p* = 0.9 (solid and empty squares). (**b**) 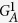 and 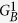 vs. *λ* for *p* = 0.1 (solid and empty circles) and *p* = 0.9 (solid and empty squares). The parameters are chosen as *L* = 100, *ρ*_0_ = 0.1 and *μ* = 0.5. The results are averaged over 10^2^ × 10^4^ independent realizations in 10^2^ different configurations of dependency links.

Figure 3 compares the dynamics of social contagion on interdependent spatial networks when *p* = 0.1 and *p* = 0.9. Figure 3(a) shows that when *p* = 0.9 the average density of final recovered nodes *R*_*A*_ in lattice *A* grows more rapidly than when *p* = 0.1. When *p* = 0.9 the behavior information from lattice *A* can easily propagates to lattice *B* because the abundant dependency links allow nodes in lattice *A* to adopt behavior through both connected infections from neighbors in the same lattice and dependency infections from the many dependent nodes in lattice *B*. The asymmetry of results in lattice *A* and *B* is due to the asymmetry of the initial condition. When *p* = 0.9 the propagation in lattice *B* is approximately the same as that in lattice *A*. When *p* = 0.1 the prevalence in lattice *B* is much lower than in lattice *A* because there are relatively few dependency links, the propagation from lattice *A* to lattice *B* is difficult, and the small number of seeds disallow outbreaks of behavior information in lattice *B*. Figure 3(b) shows the normalized sizes of the giant connected component (GCC) of final recovered nodes 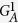 and 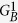 on lattices *A* and *B*, respectively. Note that the trends of the giant connected components versus the transmission probability *λ* are similar to those of the density of final recovered nodes. Unlike when *p* = 0.1, both 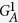 and 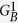 increase abruptly at some *λ* when *p* = 0.9. These results indicate that the behaviors of 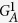 and 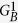 versus *λ* may be a second-order phase transition when *p* = 0.1 and a first-order phase transition when *p* = 0.9.

**Figure 4:**
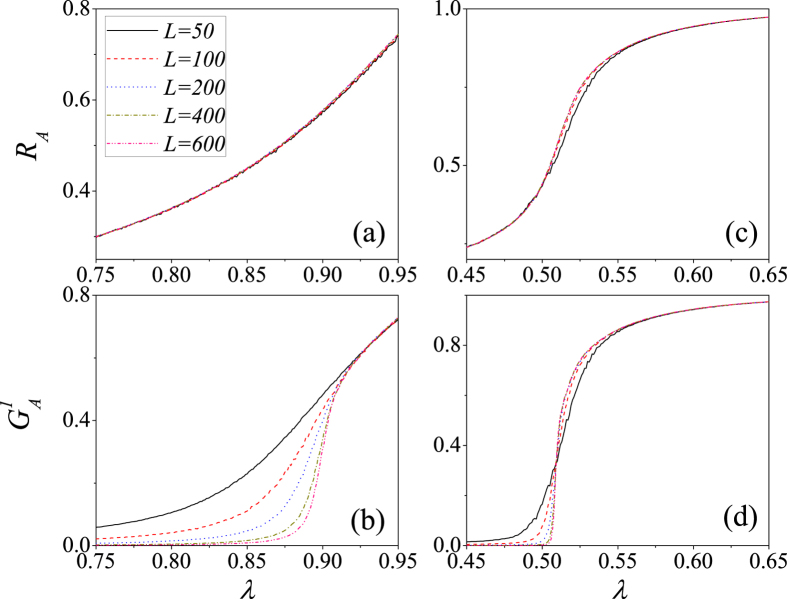
For *ρ*_0_ = 0.1, the finite-size effects on interdependent spatial networks with *p* = 0.1 (**a**,**b**) and *p* = 0.9 (**c**,**d**). (**a**) *R*_*A*_ vs. *λ* for *p* = 0.1. (**b**) 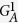 vs. *λ* for *p* = 0.1. (**c**) *R*_*A*_ vs. *λ* for *p* = 0.9. (**d**) 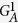 vs. *λ* for *p* = 0.9. The solid lines, dash lines, dot lines, dash dot lines and dash dot dot lines respectively represent *L* = 50, 100, 200, 400 and 600. We perform 10^2^ × 10^4^ independent realizations on 10^2^ different networks.

Figure 4 shows a finite-size analysis^47^ of lattice *A* of the type of phase transition described above. The average density of recovered nodes *R*_*A*_ are nearly the same for different linear size *L* values, especially when the interdependent network is weak [see Fig. 4(a,c)]. When *p* = 0.1, the normalized size giant connected component 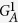 for different *L* values begin to converge after *λ* ≈ 0.915 [see Fig. 4(b)], which indicates that the behavior of GCC versus *λ* is a second-order phase transition^23^,^24^. When *p* = 0.9, all the curves intersect at one point [see Fig. 4(d)], and thus the type of phase transition will become first-order as *N* → ∞^23^,^24^. Here the abundant dependency links enable the dependent node *B*_*i*_ of an adopted node *A*_*i*_ to immediately adopt the new behavior. Node *B*_*i*_ transmits the information to one of its susceptible neighbors *B*_*u*_, which becomes adopted when the cumulative pieces of received information exceed the adoption threshold and causes the behavior to be adopted by its dependency node *A*_*u*_. This phenomenon induces cascading effects in adopting behavior, causes a large number of nodes to become adopted simultaneously, and contributes to the appearance of a first-order phase transition. These results indicate that the parameter *p* is a key factor in social contagion on interdependent spatial networks. We also perform a finite-size analysis of lattice *B* and find a similar phenomenon (see the Supplemental Material for details).

Variability methods^53^,^54^ can numerically determine the epidemic threshold^55^,^56^ in SIR epidemiological models. To determine the first-order phase transition point in a complex social contagion process, we calculate the number of iterations (NOI) required for the dynamical process to reach a steady state^16^,^24^,^57^ and count only the iterations during which at least one new node becomes adopted. For a second-order phase transition, we calculate the normalized size of the second giant connected component (SGCC) of the final recovered nodes after the dynamical process is complete^16^,^24^,^58^. In the thermodynamic limit, we obtain the second-order transition point 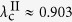 for *p* = 0.1 and the first-order transition point 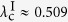 for *p* = 0.9 (see the Methods for details). We also present some critical phenomena in the Method section.

**Figure 5:**
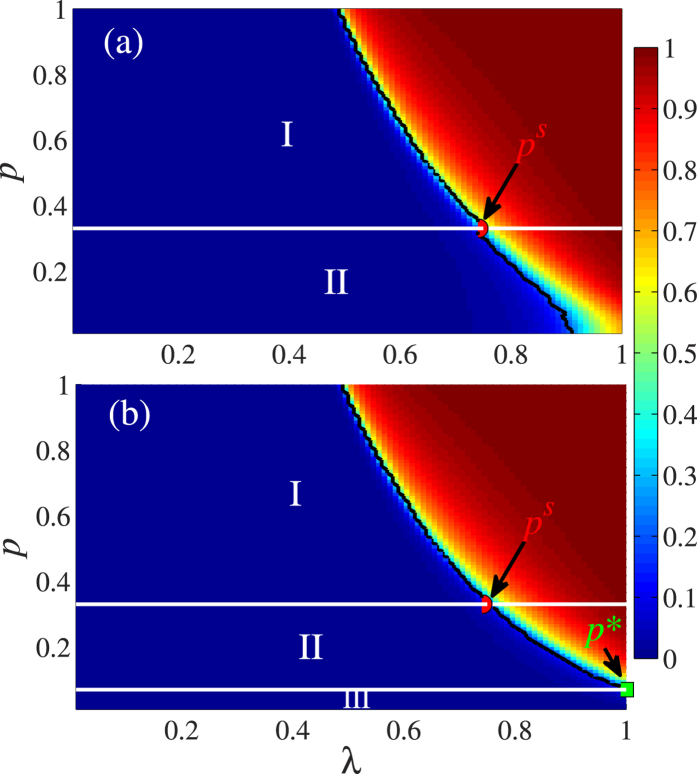
Dependency of the normalized size of giant connected components on *p* and *λ* for *ρ*_0_ = 0.1. The colors represents the normalized size of GCC. (**a**) 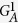 vs. *p* and *λ*. (**b**) 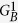 vs. *p* and *λ. p*^*s*^ indicates the critical fraction of dependency links that separates the second-order phase transition from first-order phase transition. p^*^ indicates the critical fraction of dependency links below which the behavior information could not propagate. We perform 10^2^ × 10^4^ independent realizations on 10^2^ different networks.

Figure 5 shows the dependency of 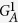 and 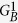 on different *p* and *λ* values. Both 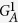 and 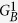 increase with *p* because many dependency links enhance the ability of the nodes to access the behavior information. Using the behavior of GCC versus *λ*, we divide the *λ* − *p* plane into different regions. Figure 5(a) shows that in lattice *A* there is a critical fraction *p*^*s*^ of dependency links that divides the phase diagram into a second-order phase transition region (region II) and a first-order phase transition region (region I). In region II most of the behavior information in lattice *A* propagates through contacts between neighbors. The dependency infection from lattice *B* is small because there are few dependency links and there is no abrupt increase of 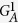 with *λ*. In region I the large number of dependency links cause cascading effects in adopting behavior, cause a large number of nodes to simultaneously become adopted nodes, and cause a first-order phase transition. In lattice *B*, the *λ* − *p* plane is divided into three different regions in which regions I and II indicate that the behaviors of GCC versus *λ* are first-order and second-order phase transitions, respectively [see Fig. 5(b)]. In contrast to lattice *A*, when *p* < p^*^ there is an additional region III within which the social behavior cannot widely propagate no matter how large the *λ* value. This is because here the few dependency links produce only a few initially-adopted nodes in lattice *B*, and they can not provide sufficient contacts with adopted neighbors for susceptible nodes to adopt the behavior. Note that both 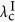 and 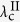 decrease as *p* increases, which indicates that the strong interdependent spatial networks are promoting the social contagion.

### Effects of the fraction of initial seeds

All of the above results depend on the initial condition in which there are *ρ*_0_ = 0.1 fraction of adopted nodes. Here we further explore the effects of the initial adopted fraction on social contagion on interdependent spatial networks.

**Figure 6:**
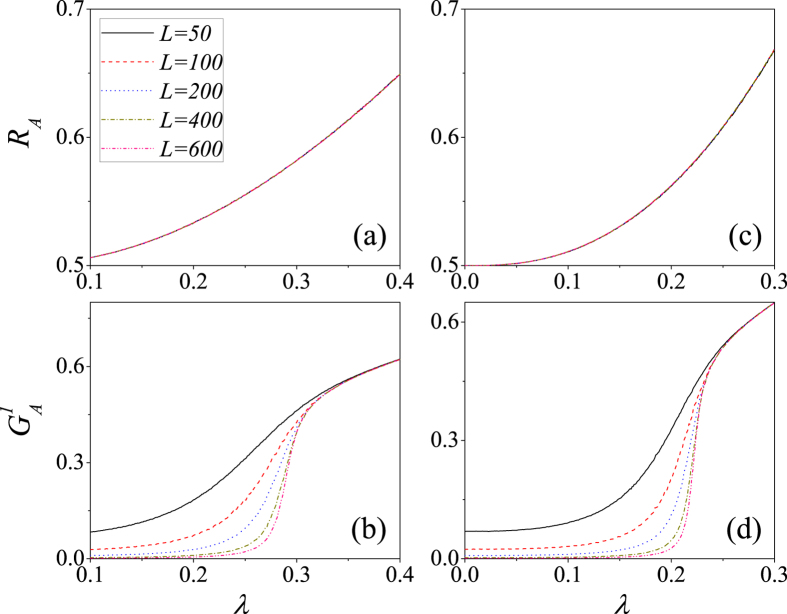
For *ρ*_0_ = 0.5, the finite-size effects on interdependent spatial networks with *p* = 0.1 (**a**,**b**) and *p* = 0.9 (**c**,**d**). (**a**) *R*_*A*_ vs. *λ* for *p* = 0.1. (**b**) 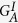 vs. *λ* for *p* = 0.1. (**c**) *R*_*A*_ vs. *λ* for *p* = 0.9. (**d**) 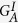 vs. *λ* for *p* = 0.9. The solid lines, dash lines, dot lines, dash-dot lines and dash-dot-dot lines respectively represent *L* = 50, 100, 200, 400 and 600. The results are averaged over 10^2^ × 10^4^ independent realizations.

Figure 6 shows the propagation when there are *ρ*_0_ = 0.5 fraction of initially-adopted nodes. Figure 6(a,c) show that *R*_*A*_ are approximately the same for different *L* values, especially when *p* = 0.1. Figure 6(b) shows that 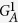 for different *L* values begin to converge after *λ* ≈ 0.334. Here the large *ρ*_0_ value provides many opportunities for susceptible nodes to receive the information. After receiving sufficient information they become adopted, and this eventually induces a second-order phase transition. Figure 6(b) shows that the analogy between *ρ*_0_ = 0.5 and *ρ*_0_ = 0.1 indicates that the type of phase transition does not change with *ρ*_0_ when *p* = 0.1. Note that all curves of 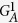 also begin to converge after *λ* ≈ 0.25 when *p* = 0.9, as shown in Fig. 6(d). This is because there are sufficient initial seeds to raise the probability of susceptible nodes becoming adopted through connected infection. The cascading effects from dependency links are somewhat weakened, and this leads to a second-order phase transition. The differences between the behaviors of 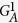 versus *λ* for *ρ*_0_ = 0.5 and *ρ*_0_ = 0.1 indicate that the phase transition is no longer first-order as *ρ*_0_ is increased when *p* = 0.9. The similar phenomena are also found in lattice *B* (see the Supplemental Material for details). According to the method of determining the second-order phase transition point, we obtain 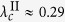 for *p* = 0.1 and 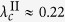 for *p* = 0.9 in the thermodynamic limit (see the Methods for details). Some critical phenomena are presented in the Method section.

**Figure 7:**
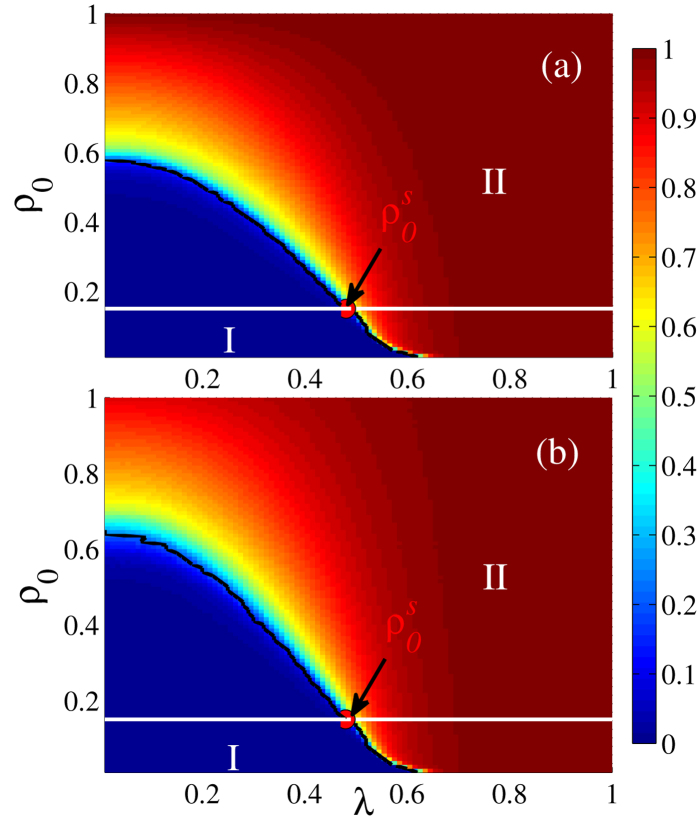
Dependency of the normalized size of giant connected component on parameters *ρ*_0_ and *λ* for *p* = 0.9. The colors represents the normalized size of GCC. (**a**) 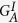 vs. *ρ*_0_ and *λ*. (**b**) 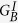 vs. *ρ*_0_ and *λ*. 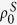 indicates the critical fraction of initial adopted nodes that separates the second-order phase transition from first-order phase transition. The results are averaged over 10^2^ × 10^4^ independent realizations.

Figure 7 shows the dependency of 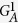 and 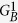 on different *ρ*_0_ and *λ* values when *p* = 0.9. Note that both 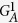 and 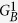 increase with *ρ*_0_ because there are many initially-adopted nodes to promote the propagation of behavior information among neighbors. Figure 7(a) uses the behavior of GCC versus *λ* to show that the phase diagram is divided into two different regions. When 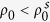, the cascading effect caused by abundant dependency links strongly promotes information propagation and leads to the first-order phase transition region (region I). When 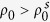, the second-order phase transition region (region II) appears, since the susceptible nodes adopt the behavior mainly through connected infection within the same lattice and the cascading effects are weakened. These phenomena indicate that on strongly interdependent spatial networks the phase transition changes from first-order to second-order as *ρ*_0_ is increased. In addition, both the second-order and first-order phase transition points decrease with *ρ*_0_. This supports the findings shown in Figs 4(d) and 6(d) and indicates the important role of the initially-adopted fraction. Figure 7(b) shows that as in lattice *A* the *λ* − *ρ*_0_ plane in lattice *B* is divided into two regions in which region I corresponds to the first-order phase transition and region II corresponds to the second-order phase transition. The phase transition points also decrease as *ρ*_0_ increases.

## Discussion

We have studied in detail the social contagion on interdependent spatial networks consisting of two finite lattices that have dependency links. We first propose a non-Markovian social contagion model in which a node adopts a new behavior when the cumulative pieces of information received from adopted neighbors in the same lattice exceed an adoption threshold, or if its dependency node becomes adopted. The effects of dependency links on this social contagion process are studied. Unlike networks with a small fraction *p* of dependency links, networks with abundant dependency links greatly facilitate the propagation of social behavior. We investigate the normalized sizes of GCC of final recovered nodes on networks of different linear sizes *L* and find that the phase transition changes from second-order to first-order as *p* increases. The first-order and second-order phase transitions points are determined by calculating the number of iterations and the normalized size of the second giant connected component, respectively. Using interdependent spatial networks, we further investigate how the fraction of initially-adopted nodes influences the social contagion process. We find that increasing the fraction of initially-adopted nodes *ρ*_0_ causes the behavior of GCC versus *λ* to change from a first-order phase transition to a second-order phase transition on networks with a large *p* value. If the *p* value of the network is small the phase transition remains second-order even when there are abundant initial seeds. In addition, both the first-order and second-order phase transition points decrease as *p* or *ρ*_0_ increases.

We have numerically studied the dynamics of social contagion on interdependent spatial networks. The results show that both the fractions of dependency links and initially-adopted node can influence the type of phase transition. Our results extend existing studies of interdependent spatial networks and help us understand phase transitions in the social contagion process. The social contagion models including other individual behavior mechanisms, e.g., limited contact ability^27^ or heterogenous adopted threshold^28^, should be further explored. Further theoretical studies of our model are very important and full of challenges since the non-Markovian character of our model and non-local-tree like structure of the lattice make it extremely difficult to describe the strong dynamical correlations among the states of neighbors.

## Methods

### Generation of the interdependent spatial networks

To establish an interdependent spatial network, we first generate two identical lattices *A* and *B* with the same linear size *L*. In each lattice all nodes are arranged in a matrix of *L* × *L*, and each node is connected to its four neighbors in the same lattice via connectivity links. We then randomly choose *p* fraction of nodes in lattice A to be dependency nodes. Once a node *A*_*i*_ in lattice *A* is chosen as a dependency node, it will be connected to one and only one node *B*_*j*_ randomly selected in lattice *B* via a dependency link [see Fig. 1(a)]. Thus, a dependency link connects two random nodes respectively located in lattice A and B with probability *p*. Each dependency node has only one dependency link. The number of dependency links in the interdependent spatial network is determined by the parameter *p*. For simplicity, the interdependent networks with a large *p* value are defined as the strong interdependent networks, and those with a small *p* value are defined as the weak ones.

### Determination of phase transition points

To locate the transition points 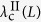 and 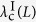 as a function of the network size *N* = *L* × *L*, we study the location of the peak of SGCC and NOI, respectively. On a network with finite size *N*, NOI reaches its peak at the first-order phase transition point and SGCC reaches its peak at the second-order phase transition point^24^. In the thermodynamic limit (i.e., *N* → ∞), the critical point 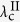 and 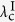 should fulfill 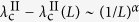 with *α* > 0 and 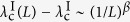 with *β* > 0, respectively^59^. Then, from the finite-size scaling theory one should obtain the scaling *G*^1^ ~ *N*^−*δ*^ (with *δ* > 0) only at the second-order phase transition point 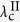, and a power law relation *NOI* ~ *N*^*γ*^ (with *γ* > 0) only at the first-order phase transition point 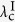.

**Figure 8:**
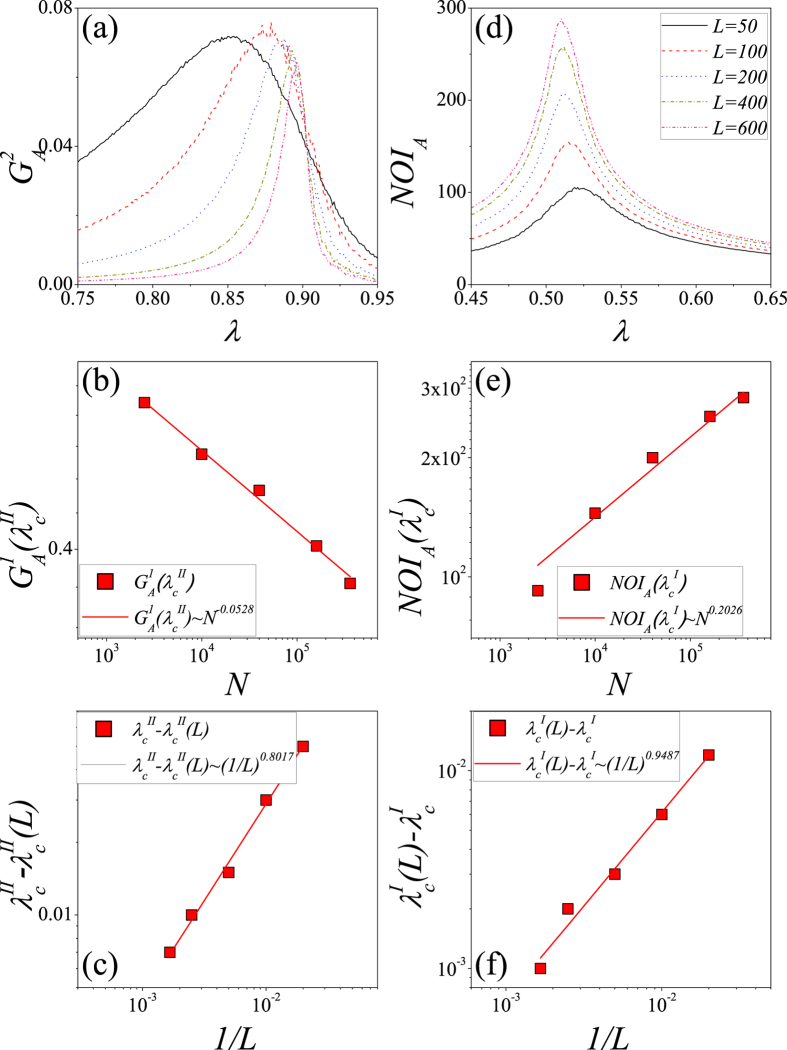
For *ρ*_0_ = 0.1, the determination of phase transition point on interdependent spatial networks with *p* = 0.1 (**a**–**c**) and *p* = 0.9 (**d**–**f**). (**a**) 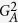 vs. *λ* for *p* = 0.1. (**b**) 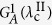 vs. *N* = *L* × *L* for *p* = 0.1. (**c**) 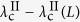 vs. 1/*L* for *p* = 0.1. (**d**) *NOI*_*A*_ vs. *λ* for *p* = 0.1. (**e**) 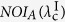 vs *N* for *p* = 0.9. (**f**) 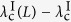 vs. 1/*L* for *p* = 0.9. In figures (**a**,**d**), the solid lines, dash lines, dot lines, dash dot lines and dash dot dot lines respectively represent *L* = 50, 100, 200, 400 and 600. We perform 10^2^ × 10^4^ independent realizations on 10^2^ different networks.

Figure 8(a) shows that when *p* = 0.1, the peak of the normalized size of the second giant connected component in lattice *A* (i.e., 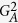) versus *λ* gradually shifts to the right as *L* is increased. In Fig. 8(b) we plot 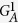 versus *N* = *L* × *L* for fixed *λ*. We obtain a power law relation 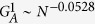 at 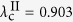. Then we fit 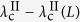 versus 1/*L* by using the least-squares-fit method in Fig. 8(c). We find that 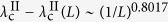. Figure 8(d) shows that when *p* = 0.9, the peak of NOI in lattice *A* (i.e., *NOI*_*A*_) versus *λ* gradually shifts to the left as *L* is increased. In Fig. 8(e) we plot *NOI*_*A*_ versus *N* for fixed *λ*, and obtain a power law relation *NOI*_*A*_ ~ *N*^0.2026^ at 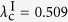. We further fit 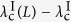 versus 1/*L* by using the least-squares-fit method in Fig. 8(f), and find that 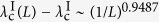.

**Figure 9:**
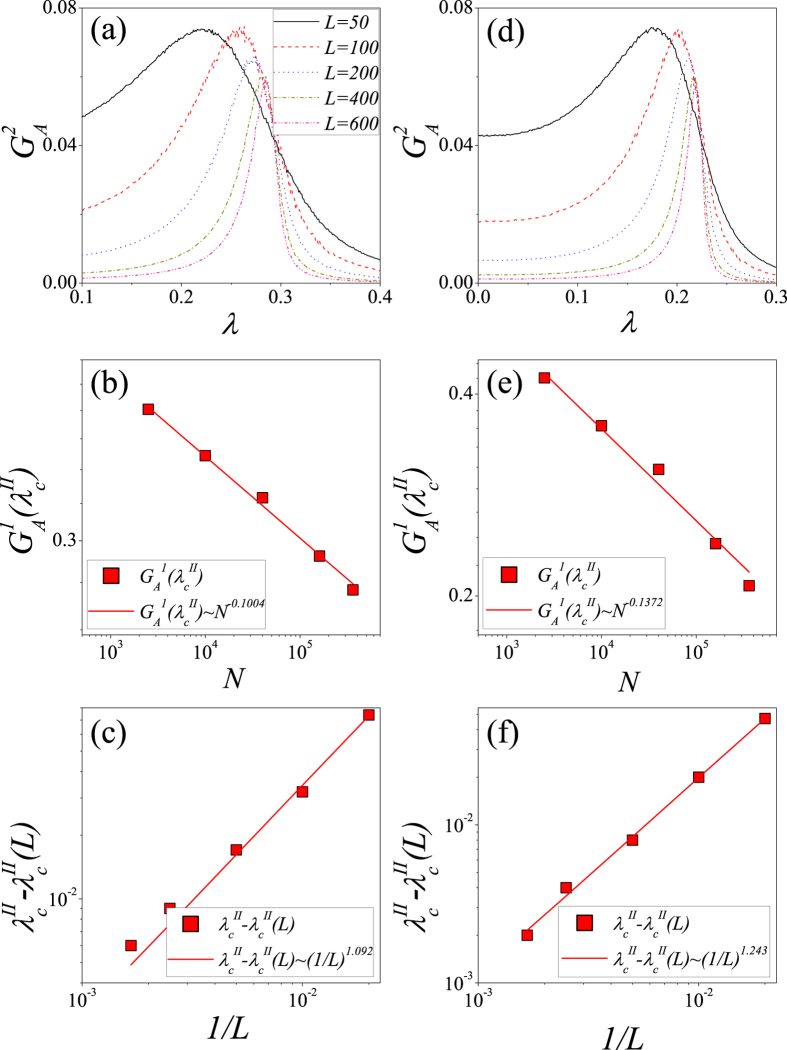
For *ρ*_0_ = 0.5, the determination of phase-transition point on interdependent spatial networks with *p* = 0.1 (**a**–**c**) and *p* = 0.9 (**d**–**f**). (**a**) 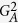 vs. *λ* for *p* = 0.1. (**b**) 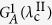 vs. *N* = *L* × *L* for *p* = 0.1. (**c**) 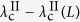 vs. 1/*L* for *p* = 0.1. (**d**) 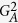 vs. *λ* for *p* = 0.9. (**e**) 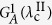 vs. *N* = *L* × *L* for *p* = 0.9. (**f**) 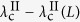 vs. 1/*L* for *p* = 0.9. In figures (**a**,**d**), the solid lines, dash lines, dot lines, dash-dot lines and dash-dot-dot lines respectively represent *L* = 50, 100, 200, 400 and 600. The results are averaged over 10^2^ × 10^4^ independent realizations.

We perform the similar analyses for *ρ*_0_ = 0.5, as shown in Fig. 9. Figure 9(a) shows that when *p* = 0.1, the peak of 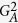 versus *λ* gradually shifts to the right as *L* is increased. In Fig. 9(b) we plot 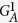 versus *N* = *L* × *L* for fixed *λ*. We obtain a power law relation 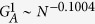 at 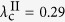. Then we fit 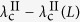 versus 1/*L* in Fig. 9(c). We find that 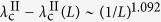. Fig. 9(d) shows that when *p* = 0.9, the trend of 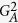 versus *λ* as *L* is increased is similar to that when *p* = 0.1. In Fig. 9(e) we plot 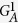 versus *N* = *L* × *L* for fixed *λ*, and obtain a power law relation 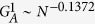 at 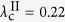. We further fit 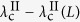 versus 1/*L* in Fig. 9(f), and find that 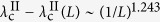.

## Additional Information

**How to cite this article:** Shu, P. *et al*. Social contagions on interdependent lattice networks. *Sci. Rep.* **7**, 44669; doi: 10.1038/srep44669 (2017).

**Publisher's note:** Springer Nature remains neutral with regard to jurisdictional claims in published maps and institutional affiliations.

## Supplementary Material

Supporting Information
