# Supplementary material for: Social contagions on interdependent lattice networks
Source: Sci Rep. 2017 Mar 16;7:44669. doi: 10.1038/srep44669 (PMC5353708; doi:10.1038/srep44669)
Supplement: Supporting Information [file srep44669-s1.pdf]

# Supporting information for 'Social contagions on interdependent lattice networks'


Panpan Shu,[1] Lei Gao,[2] Pengcheng Zhao,[3] Wei Wang,[2,4,5,*] and H. Eugene Stanley[5]

[1]*School of Sciences, Xi'an University of Technology, Xi'an 710054, China*
[2]*Web Sciences Center, University of Electronic Science and Technology of China, Chengdu 610054, China*
[3]*School of Physics and Optoelectronic Engineering, Xidian University, Xi'an 710071, China*
[4]*Big data research center, University of Electronic Science and Technology of China, Chengdu 610054, China*
[5]*Center for Polymer Studies and Department of Physics, Boston University, Boston, Massachusetts 02215, USA*



(Dated: January 15, 2017)


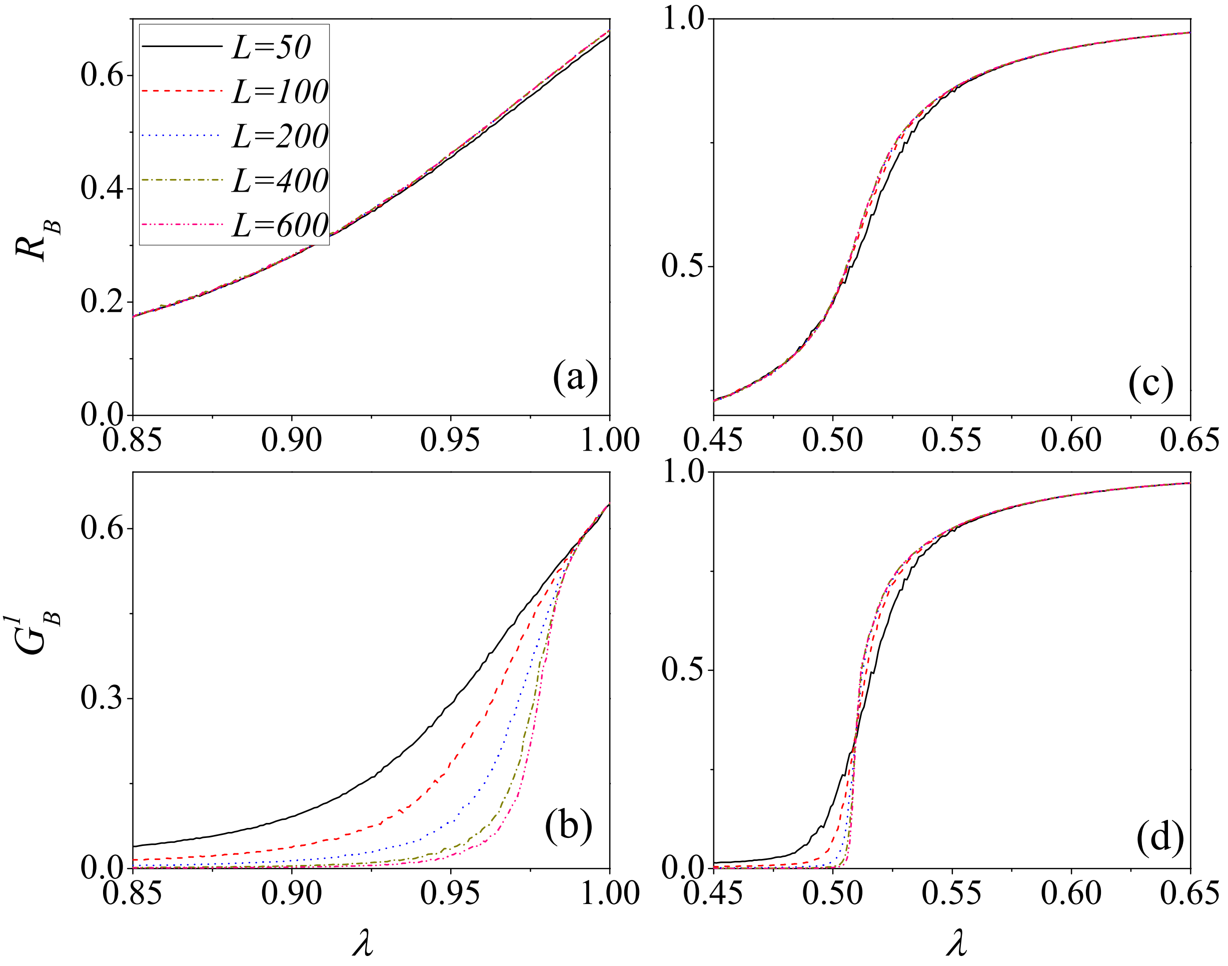


FIG. S 1: (Color online) For $\rho_0 = 0.1$, the finite-size effects on interdependent spatial networks with $p = 0.1$ (a-b) and $p = 0.9$ (c-d). (a) $R_B$ vs. $\lambda$ for $p = 0.1$. (b) $G_B^1$ vs. $\lambda$ for $p = 0.1$. (c) $R_B$ vs. $\lambda$ for $p = 0.9$. (d) $G_B^1$ vs. $\lambda$ for $p = 0.9$. The solid lines, dash lines, dot lines, dash dot lines and dash dot dot lines respectively represent $L = 50, 100, 200, 400$ and 600. We perform $10^2 \times 10^4$ independent realizations on $10^2$ different networks.


*Electronic address: `wwzqbx@hotmail.com`

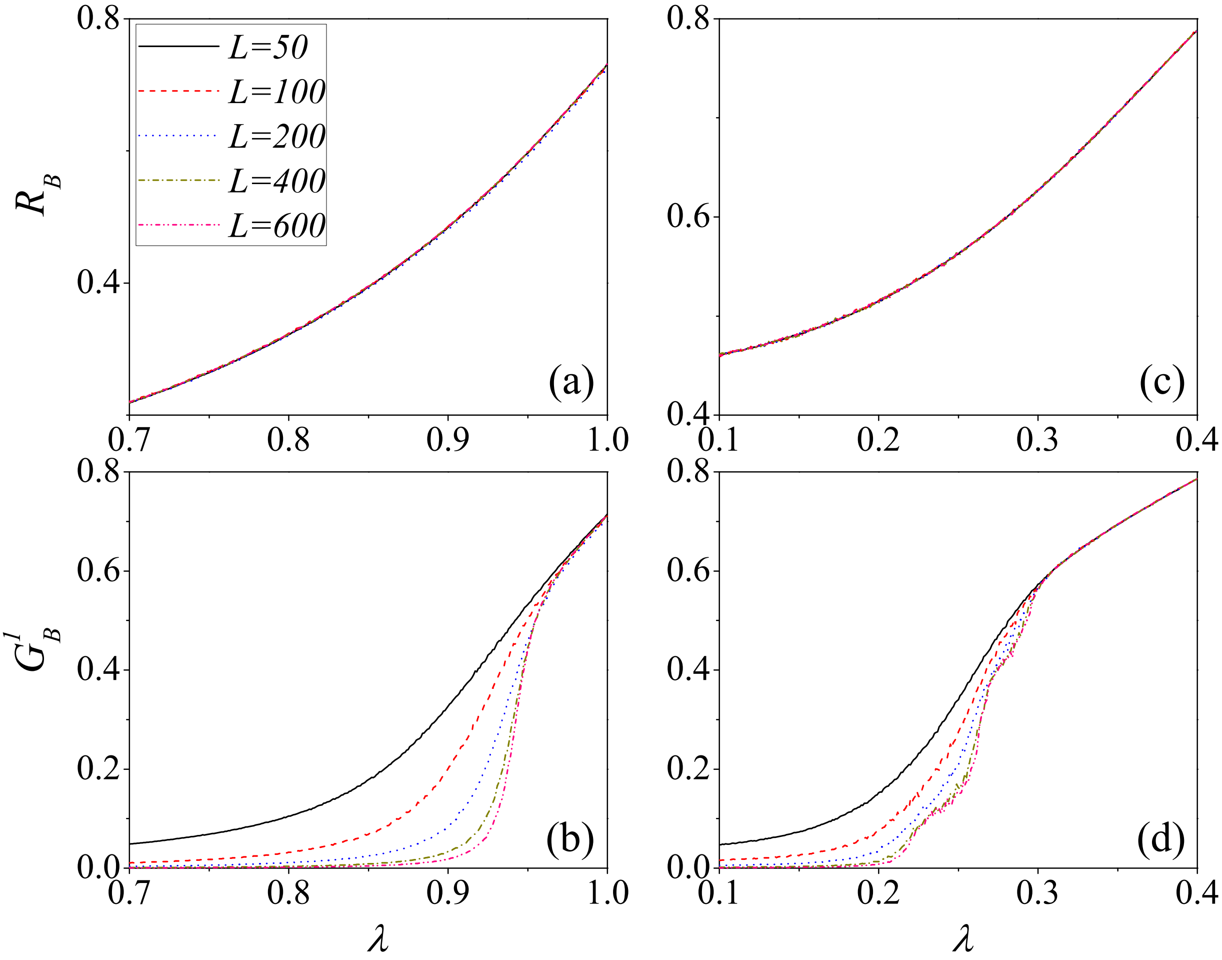


FIG. S 2: (Color online) For $\rho_0 = 0.5$, the finite-size effects on interdependent spatial networks with $p = 0.1$ (a-b) and $p = 0.9$ (c-d). (a) $R_B$ vs. $\lambda$ for $p = 0.1$. (b) $G_B^1$ vs. $\lambda$ for $p = 0.1$. (c) $R_B$ vs. $\lambda$ for $p = 0.9$. (d) $G_B^1$ vs. $\lambda$ for $p = 0.9$. The solid lines, dash lines, dot lines, dash dot lines and dash dot dot lines respectively represent $L = 50, 100, 200, 400$ and 600. We perform $10^2 \times 10^4$ independent realizations on $10^2$ different networks.
